# TLR7 Polymorphism (rs179008 and rs179009) in HIV-Infected Individual Naïve to ART

**DOI:** 10.1155/2020/6702169

**Published:** 2020-05-20

**Authors:** HariOm Singh, Dharmesh Samani, Sumit Aggarwal

**Affiliations:** ^1^Department of Molecular Biology, National AIDS Research Institute Pune, 411026, India; ^2^Division of ECD, Indian Council of Medical Research-HQ, New Delhi 110029, India

## Abstract

Toll-like receptors (TLRs) play an important role in the innate immune response to HIV infection. Single nucleotide polymorphism (SNP) in *TLR7* (Gln11Leu) gene has been associated with a rapid decline of CD4T cell count. Hence, we assessed the *TLR7* (rs179008, Gln11Leu (A/T) and rs179009, IVS2-151 (A/G)) polymorphism in 150 HIV-infected individuals naïve to ART and 158 healthy controls. The genotyping of *TLR7* Gln11Leu (A/T) and IVS2-151 (A/G) polymorphisms was done using the PCR-RFLP method. In univariate analysis, none of the genotype and haplotype of *TLR7* Gln11Leu (A/T) and IVS2-151 (A/G) polymorphism differed significantly between HIV-infected individuals and healthy controls. The occurrence of *TLR7* rs179009AG genotype in the codominant model and rs179009 AG-GG genotype in the dominant model was significantly reduced in HIV-infected individuals as compared to healthy controls (18.0% vs. 29.1%, OR = 0.42, *P* = 0.016; 26.7% vs. 36.7%, OR = 0.52, *P* = 0.016). TLR7 rs179009AG genotype was significantly underrepresented in the intermediate HIV disease stage compared with healthy controls (OR = 0.03, *P* = 0.04). TLR7 rs179009AG genotype expressed higher in tobacco-consuming HIV-infected individuals compared with nonusers (OR = 1.71, *P* = 0.47). In conclusion, rs179009 AG-GG and AG genotypes were found reduced in HIV-infected individuals as compared to healthy controls; their higher prevalence in health individuals clearly support that they are associated with reduced risk of acquisition of HIV-1 infection.

## 1. Introduction

In India, HIV remains to be one of the major issues (WHO, HIV/AIDS Fact sheet November 2017). The difference in susceptibility to the acquisition of HIV and disease progression is associated with a genetic background [1]. Innate immunity is known to play a crucial role in response to viral pathogens [2]. Toll-like receptors (TLRs) are pathogen recognition receptors (PRRs) that play a pivotal role in the innate immune system and control inflammatory responses and adaptive immunity [3]. PRRs recognize evolutionarily conserved pathogen-associated molecular patterns (PAMPs), and PAMP recognition triggers activation of signal transduction pathways and downstream effector responses [4, 5]. TLR families like TLR1, TLR2, TLR4, and TLR6 are localized on the cell surface and TLR3, TLR7, TLR8, and TLR9 are localized in endosomal membranes inside the cell [4, 5].

*TLR7* plays an important role in an antiviral immune response. *TLR7* recognizes RNA of various viruses including HIV [6]. *TLR7* bind single-stranded RNA [7]. It shares a high degree of structural similarity, and their activation leads to nuclear factor-kappa B (NF-*κ*B) activation and subsequent production of inflammatory cytokines [8]. The *TLR7* gene span 16 kb apart, located on the Xp22.2 chromosome [9]. Single nucleotide polymorphisms (SNPs) in TLR genes may contribute to differences in the acquisition of HIV-1 and disease progression [6]. Some of the studies reported that the functional polymorphism of *TLR7* rs179008, Gln11Leu was associated with HIV acquisition [10], higher viral loads and accelerated progression to advanced immune suppression in HIV patients [6], hepatitis C infection [11], basal cell carcinoma (BCC) [12], female HCV patients, and spontaneous clearance of HCV [13]. Elefanti et al., [14] have been reported that *TLR7* 32A/T polymorphism was not associated with melanoma cases. Similarly, *TLR7* rs179009, IVS2-151A/G polymorphism was associated with spontaneous clearance of HCV in female patients [13] and chronic hepatitis C [15]. Wang et al., [16] reported the frequency of IVS2-151A allele (77%) was higher as compared to IVS2-151G (22%). *TLR7* 32A/T polymorphism was associated with HIV-1 disease progression in HIV-1-infected women compared to uninfected women [6]. TLR7 rs5743737 and *TLR7* rs1634323 polymorphisms were associated with a decreased risk of BV while *TLR7* rs179012 was associated with an increased risk [17].

Till now, none of the studies reported the association of *TLR7* polymorphisms (rs179008 and rs179009) with the HIV infection and disease progression from India. Hence, we attempted to assess the distribution of *TLR7* polymorphisms (rs179008 and rs179009) in HIV-infected individuals naïve to ART from the Western Indian population.

## 2. Materials and Methods

### 2.1. Subjects

The subject recruitment period was April 2014 to March 2017; 153 HIV-infected individuals naive to ART, aged between 18 and 50 years, were consecutively taken from the outdoor patient clinics of the National AIDS Research Institute, Pune. At the same time, 158 individuals free of (individuals from the same family excluded) HIV, hepatitis B and C, and tuberculosis were age-matched and serum negative from ELISA tests were recruited from the clinic belonging to the same institution. Clinical data was collected via a questionnaire, personal interviews, and review of case records. The estimation of CD4 count was done by fluorescence-activated cell sorting (FACS). CD4+ count < 350 cells/mm^3^, 350-500 cells/mm^3^, and >500 cells/mm^3^ were considered as advanced, intermediate, and early stage of HIV infection. ELISA for hepatitis C and HBsAg testing was performed using the Ortho HCV ELISA test system and Murex HBsAg confirmatory (DiaSorin) ELISA. Environmental exposures (tobacco and alcohol consumption) were also recorded in the questionnaire. The study was approved by local ethics committees of the institute, and all participants gave informed signed consent. Two ml of the peripheral blood sample was collected and stored at -700°C before DNA extraction. Genomic DNA extraction was done from peripheral blood leukocyte pellet using the AxyPrep Blood Genomic DNA Miniprep Kit according to the protocol given by the manufacturer.

### 2.2. Genotyping

Single nucleotide polymorphisms (rs179008 and rs179009) of the *TLR7* gene were genotyped in subjects using polymerase chain reaction-restriction fragment length polymorphism (PCR-RFLP).

The primer for the amplification of TLR7 polymorphisms was used as reported by [18]. The primer sequence of *TLR7* rs179008 and rs179009 polymorphisms was F: 5′-TAACAACGAATAGG AAAATGC-3′ and R: 5′-GTTTTAGGAAACCATCTAGCC-3′ [18]. A total volume of 25 *μ*l with 20 pmol of each primer, genomic DNA (100-150 ng), 10 mM deoxynucleotide triphosphates, PCR buffer containing 100 mM Tris-HCl, pH 8.6, 50 mM KCl, 1.5 mM MgCl_2_, and 1.5 units of Taq polymerase (Bangalore Genei, India) was used for PCR. The reaction conditions for *TLR7* polymorphisms (rs179008) were initial denaturation at 94°C for 5 min, followed by 35 cycles of denaturation at 94°C for 35 sec, annealing at 54°C for 30 sec, extension at 72°C for 1 min, and final extension at 72°C for 5 min. Amplified products of rs179008 and rs179009 polymorphisms were digested using restriction enzyme ApoI and NlaIII (MBI Fermentas Inc., USA), respectively. Genotyping of *TLR7* polymorphisms was done in 15% polyacrylamide gel using molecular weight markers and visualized after staining with ethidium bromide. Based on sequences and location of SNP, *TLR7* polymorphism rs179008 A/T was genotyped as assigned as follows: 166 bp + 37 bp + 66 bp for AA, 203 bp + 166 bp + 37 bp + 66 bp for AT, and 203 bp + 66 bp for TT. *TLR7* rs179009 A/G was genotype as 122 bp + 247 bp for AA, 122 bp + 247 bp + 369 bp for AG, and 369 bp for GG. Veriti 96 well thermal cycler was used for amplification of reactions (Applied Biosystems, USA). PCR products and molecular weight markers were visualized after staining with ethidium bromide. Twenty percent of samples from both patients and controls were regenotyped by other laboratory personnel, and no discrepancy in genotyping was noticed. Sequencing was done in 10% of samples to assess the genotyping error.

### 2.3. Data Analysis

The age variable was expressed as a mean ± standard deviation (SD). The *χ*^2^ goodness-of -fit test was used for any deviation from Hardy-Weinberg equilibrium in controls. We used the *χ*^2^ statistic (Fisher's exact test for cell size < 5) to compare genotype frequency between HIV-infected individuals vs. healthy controls. SNPStats software was used to compare haplotype frequency between HIV-infected individuals and controls. Interaction of tobacco use, alcohol intake, and genotypes was examined in all eligible HIV-infected individuals. Odds ratios (ORs) and 95% confidence interval (CI) were calculated by unconditional binary logistic regression. All statistical analysis was performed using SPSS software version 17.0 (SPSS, Chicago, IL, USA), and tests of statistical significance were two-sided and taken as significant when *P* value was less than 0.05.

## 3. Results

In the present study, we enrolled 150 HIV-infected individuals and 158 healthy controls. The mean age (years ± SD) of HIV-infected individuals and healthy controls were 36 yrs ± 3.2 and 33 yrs ± 6.47, respectively. The demographic profile of HIV-infected individuals and healthy controls is shown in Table 1.

### 3.1. Genotype-Phenotype Association

#### 3.1.1. TLR7 (rs179008 and rs179009) Polymorphism and HIV-Infected Individuals

The occurrence of *TLR7* polymorphism in HIV-infected individuals and healthy controls is shown in Table 2. Polymorphisms rs179008 and rs179009 in *TLR7* gene followed the Hardy-Weinberg equilibrium (*P* = 0.07, 0.05) in healthy controls. The distribution of rs179009 AG genotype in the codominant model, rs179009 AG-GG genotype in the dominant model, and rs179009 AG genotype in the overdominant model was significantly reduced in HIV-infected individuals as compared to healthy controls (18.0% vs. 29.1%, OR = 0.42, 95% CI: 0.23-0.77, *P* = 0.016; 26.7% vs. 36.7%, OR = 0.52, 95% CI: 0.30-0.89, *P* = 0.016, and 18% vs. 29.1%, OR = 0.42, 95% CI: 0.23-0.77, *P* = 0.0039).

#### 3.1.2. TLR7 Haplotypes in HIV-Infected Individuals

The distribution of *TLR7* haplotypes (rs179008 and rs179009) in HIV-infected individuals and healthy controls is shown in Table 3. In both polymorphisms, a nonsignificant association was observed in the linkage disequilibrium (LD) (*D*′) between HIV-infected individuals vs. healthy controls (*D*′: 0.2663). Haplotype AG (rs179008∗A/rs179009∗G) was found to be higher in HIV-infected individuals as compared to healthy controls (7.00% vs. 5.41%, OR = 1.35, 95% CI: 0.64-2.84, *P* = 0.43). Haplotypes AA, TA, and TG (rs179008∗A,∗T,∗T/rs179009∗A,∗A,∗G) were expressed almost similarly (75.33% vs. 72.44%, 14.33% vs. 16.8%, and 3.33% vs. 5.35%) (Table 3).

#### 3.1.3. TLR7 Polymorphism and HIV Disease Stages

The prevalence of *TLR7* rs179009 AG genotype was observed less in early, intermediate, and advanced HIV disease stage individuals as compared to healthy controls (5.0% vs. 29.1%, OR = 0.11, 95% CI: 0.02-0.52, *P* = 0.001; 5.7% vs. 29.1%, OR = 0.03, 95% CI: 0.11-0.96, *P* = 0.04; and 4.7% vs. 29.1%, OR = 0.11, 95% CI: 0.03-0.39, *P* ≤ 0.001). Frequency of *TLR7* rs179009 GG genotype was reduced in advanced HIV disease stage as compared with healthy controls (1.6% vs. 7.6%, OR = 0.14, 95% CI: 0.01-1.07, *P* = 0.03) (Table 4).

### 3.2. Gene-Environment Interaction

#### 3.2.1. TLR7 Polymorphism and Environmental Factors

Consumption of tobacco and alcohol in HIV-infected individuals was analyzed for *TLR7* polymorphism (Table 5). *TLR7* (rs179008 and rs179009) polymorphisms did not differ between HIV-infected individuals with and without tobacco and alcohol users. The distribution of *TLR7* rs179009AG genotype was found to be higher in tobacco-consuming HIV-infected individuals as compared to nonusers (26.1% vs. 17.2%, OR = 1.71, 95% CI: 0.52-5.49, *P* = 0.47). The prevalence of TLR7 rs179008 CC, CT, and TT genotypes was almost corresponding between tobacco-consuming HIV-infected individuals and nonconsuming (82.6% vs. 82.8%, 13.0% vs. 13.9%, and 4.3% vs. 3.3%). *TLR7* rs179008 AA, AT, and TT and rs179009 AA, AG and GG genotypes distributed almost alike between alcohol taking HIV-infected individuals and nonusers (87.0% vs. 81.9%, 13.0% vs.14.2%, and 0.0% vs.3.9% and 78.3% vs. 72.4%, 17.4% vs. 18.1%, and 4.3% vs. 9.4%).

#### 3.2.2. Multivariate Logistic Regression Analysis

Correlation of age, sex, tobacco, alcohol, baseline CD4 counts, and *TLR7* (rs179008 and rs179009) polymorphisms with HIV disease progression was done by multivariate logistic regression analysis. *TLR7* (rs179008 and rs179009) polymorphisms, age, sex, tobacco, alcohol usage, and baseline CD4 counts were not found as an independent risk factor for HIV disease progression (table not shown).

## 4. Discussion

From India, this is a first of its kind study that examined the likely effects of *TLR7* (rs179008 and rs179009) polymorphisms on susceptibility to the acquisition of HIV-1 and disease progression. The genetic background of human populations can influence the susceptibility and outcome of infectious diseases. Reports suggested that the variations within the host's genome play a role in acquiring HIV infection and disease progression [19–24]. *TLR* gene variants and downstream signaling molecules could influence the ability of the affected individual to respond to *TLR* ligands resulting in altered susceptibility to or course of infectious disease [25–27]. *TLR7* polymorphism plays a role in HIV disease progression and viral load [28].

In our study, the prevalence of *TLR7* rs179008 AA, AT, and TT genotypes in healthy controls was 81.0%, 16.5%, and 2.5%. This finding was unmatched with that of the other studies carried out by [12, 13, 29, 14]. The occurrence of *TLR7* rs179008A/T allelic frequency was comparable with the study carried out by Valverde-Villegas et al., 2017, [30, 6, 16] and dissimilar with the study reported by [6, 12, 13, 29]. The occurrence of *TLR7* rs179009 polymorphism was matched with studies published by Wang et al., [16] and unmatched with studies published by. We had observed a nonsignificant distribution of *TLR7* rs179008 polymorphism between HIV-infected individuals and healthy controls, although *TLR7* rs179008AT genotype was much more prevalent in HIV-infected individuals as compared to healthy controls (3.3% vs. 2.5%, OR = 1.54). While as an association was seen between *TLR7* rs179009AG genotype and acquisition of HIV-1 (OR = 0.42, *P* = 0.016), *TLR7*rs179008A/T polymorphism was associated with susceptibility to asthma [17], course of hepatitis C infection [12], HIV-1 acquisition, and disease progression [6]. *TLR7* rs179008 polymorphism was not associated with patients of basal cell carcinoma (BCC) [12], melanoma cases (*P* = 0.245), and between single melanoma and MPM cases (*P* = 0.482) [14]. *TLR7* IVS2-151G/A allele was associated with the protection of female patients of chronic hepatitis C (*P* ≤ 0.001, OR =0.46) [15]. The finding of our study is supported by Wei et al., [15]. TLR7 IVS2-151GG genotype and IVS2-151G allele were associated with female HCV patients (*P* = 0.03 and ≤0.001) [31]. *TLR7* rs179008 (Gln11Leu) and rs179009 (IVS2-151A/G) polymorphisms were associated with the spontaneous clearance of HCV [13].

We also analyzed the haplotype to evaluate the synergistic role of two SNPs (rs179008 and rs179009) in the *TLR7* gene cluster. The occurrence of *TLR7* haplotype AG (rs179008∗A/rs179009∗G) had increased in HIV-infected individuals as compared with healthy controls (7.00% vs. 5.41%, OR = 1.35).

We undertake a case-control study, CD4+ cell count used as a surrogate marker for HIV-1 infection. The timing for the acquisition of HIV-1 was unknown; hence, the effects may have had confounding consequences. Here, we analyzed the association of *TLR7* (rs179008 and rs179009) polymorphisms with HIV disease stages to reveal the impact of *TLR7* genotypes in the risk of HIV disease advancement. *TLR7* rs179009AG genotype was associated with the early HIV disease stage individuals as compared with healthy controls (OR = 0.11, *P* = 0.001). The occurrence of the *TLR7* rs179009 GG genotype was reduced in advanced HIV disease stage as compared with healthy controls (1.6% vs. 7.6%, OR = 0.14, *P* = 0.03). *TLR7* rs179008 Gln11Leu polymorphism was associated with higher viral loads and accelerated progression to advanced immune suppression in HIV patients [6].

Most diseases are a consequence of a multifaceted interaction between an individual's genetic background and the environmental factors that individuals are exposed to. Gene-environment interaction describes the disease risk with individuals of different genotypes [32, 33]. Here, a case-only method is being adopted to evaluate gene-environment interaction. It is considered a better method. For a case-control association study of environmental influences, cases must have matched controls in the population [34]. Therefore, we used a case-only study to evaluate the risk of HIV disease progression in alcohol and tobacco users. Individuals with HIV infection and excessive alcohol consumption had a negative impact on the CD4 cell count but not on combined antiretroviral therapy [35]. In a study, a decreased response to antiretroviral therapy was observed in women who were smoking and having HIV infection [36]. *TLR7* rs179009AG genotype showed a risk for HIV disease progression among tobacco-consuming HIV patients (26.1% vs. 17.2%, OR = 1.71). However, the risk could not reach significance. This suggested that HIV-infected individuals consuming tobacco with *TLR7* rs179009AG genotypes are more prone to HIV disease progression.

The present study has certain limitations. (1) It can address the association. (2) It cannot establish causation. (3) We did not study the effect of other region polymorphisms of *TLR7* gene on HIV infection and disease progression due to lack of funds. (4) We had not addressed the DNA sequences corresponding to the expression of mRNAs coding for variants and the actual proteins responsible in the cells due to budgetary constraints. (5) The control population was not exposed to HIV. (6) The data for viral load and other clinical manifestations were not recorded in our clinical research proforma (CRF).

In summary, overall, *TLR7* rs179008 polymorphisms did not correlate with susceptibility to the acquisition of HIV and disease progression. TLR7 rs179009AG genotype could be protective for the acquisition of HIV-1. However, *TLR7* rs179009A/G polymorphism could assist in disease progression among tobacco users. *TLR7* rs179008 TT genotype in individuals with early HIV disease and rs179009 AG genotype in individuals with advanced HIV disease could protect the advancement of HIV disease. As *TLR7* is an important player to trigger the IFN-*γ* production, which exerts a direct antiviral effect through the inhibition of viral replication, it is also involved in the progression of HIV infection to AIDS. The present study should be validated with a bigger sample size in populations of other regions of India, since a limited study has been done concerning the influence of *TLR7* rs179009A/G polymorphism on HIV infection and disease progression. Hence, to better understand the role of *TLR7* rs179009A/G polymorphism in susceptibility to HIV infection and disease progression, further studies are needed.

## Figures and Tables

**Table 1:** Characteristics of HIV-infected individuals and healthy controls.

Subjects	HIV-infected individuals	Healthy controls
Number	150	158
Mean age (range)	36 ± 3.2 (years ± SD)	33 ± 6.47 (years ± SD)
Sex		
Females	76 (50.07%)	41 (25.09%)
Males	74 (49.03%)	117 (74.01%)
Alcohol habit		
User	18 (11.76%)	23 (14.74%)
Nonuser	90 (58.82%)	133 (85.25%)
Tobacco habit		
User	22 (14.37%)	22 (14.10%)
Nonuser	86 (56.20%)	134 (85.89%)
CD4+ status		
0-350	68 (44.44%)	NA
351-500	41 (26.79%)	NA
501 above	44 (28.75%)	NA

0 = data was not recorded in questionnaire form; NA = not applicable.

**Table 2:** Frequency distribution of genotypes/alleles and risk estimates of *TLR7* rs179008, *Gln11Leu* (*A/T*) and rs179009, IVS2-151(A/G) polymorphisms in the HIV-infected individuals and healthy controls.

Model	Genotypes *TLR7* rs179008, *A/T*	HIV-infected individuals *N* = 150 (%)	Healthy controls *N* = 158 (%)	*P* value	OR (95% CI)

Codominant	AA	124 (82.7%)	128 (81.0%)	1	Reference
	AT	21 (14.0%)	26 (16.5%)	0.85	0.90 (0.45-1.81)
	TT	5 (3.3%)	4 (2.5%)	0.59	1.54 (0.31-7.60)
Allele	*TLR7* rs179008 *A/T* allele	*N* = 300 (%)	*N* = 316 (%)	*P* value	OR (95% CI)
	A	269 (89.66%)	282 (89.24%)	1	Reference
	T	31 (10.33%)	34 (10.75%)	0.96	0.96 (0.55-1.65)
Dominant	AA	124 (82.7%)	128 (81%)	1	Reference
	AT-TT	26 (17.3%)	30 (19%)	0.94	0.98 (0.52-1.85)
Recessive	AA-AT	145 (96.7%)	154 (97.5%)	1	Reference
	TT	5 (3.3%)	4 (2.5%)	0.62	1.42 (0.34-5.92)
Overdominant	AA-TT	129 (86%)	132 (83.5%)	1	Reference
	AT	21 (14%)	26 (16.5%)	0.75	0.89 (0.45-1.79)

Model	Genotypes *TLR7* rs179009 A/G	HIV patients *N* = 153 (%)	Healthy controls *N* = 158 (%)	*P* value	OR (95% CI)

Codominant	AA	110 (73.3%)	100 (63.3%)	1	Reference
	AG	27 (18.0%)	46 (29.1%)	0.016	0.42 (0.23-0.77)
	GG	13 (8.7%)	12 (7.6%)	0.86	0.97 (0.40-2.43)
Allele	Allele *TLR7* rs179009 A/G	*N* = 306 (%)	*N* = 316 (%)	*P* value	OR (95% CI)
	A	247 (80.71%)	246 (77.84%)	1	Reference
	G	53 (17.32%)	70 (22.15%)	0.19	0.75 (0.50-1.14)
Dominant	AA	110 (73.3%)	100 (63.3%)	1	Reference
	AG-GG	40 (26.7%)	58 (36.7%)	0.016	0.52 (0.30-0.89)
Recessive	AA-AG	137 (91.3%)	146 (92.4%)	1	Reference
	GG	13 (8.7%)	12 (7.6%)	0.68	1.21 (0.48-3.07)
Overdominant	AA-GG	123 (82%)	112 (70.9%)	1	Reference
	AG	27 (18%)	46 (29.1%)	0.003	0.42 (0.23-0.77)

N = number of subjects; % = frequency of genotypes/alleles. The presence of AA for AT, TT for rs179008, AA for AG, GG for rs179009, and A allele for T and A allele for G was taken as a reference group for statistical analysis.

**Table 3:** Frequency distribution of haplotypes of *TLR7* polymorphisms (rs179008, *Gln11Leu* (*A/T*) and rs179009, IVS2-151 (A/G)) in HIV-infected individuals and healthy controls.

Haplotypes *TLR7* (rs179008 and rs179009)	HIV-infected individuals *N* = 300 (%)	Healthy controls *N* = 316 (%)	*P* value	OR (95% CI)
AA	75.33%	72.44%	—	Reference
AG	14.33%	16.8%	0.22	0.75 (0.48-1.18)
TA	7.00%	5.41%	0.43	1.35 (0.64-2.84)
TG	3.33%	5.35%	0.32	0.66 (0.29-1.50)

∗*N* = number of chromosomes; % = haplotype frequency; age-adjusted odds ratios, and 95% CIs were derived from logistic regression models comparing the haplotype AA with other haplotypes.

**Table 4:** Frequency distribution of *TLR7* polymorphisms (rs179008, *Gln11Leu* (*A/T*) and rs179009, IVS2-151 (A/G) in HIV disease stages and healthy controls.

*TLR7* rs179008 A/T genotypes	Healthy controls *N* = 158 (%)	Early HIV disease stage		Intermediate HIV disease stage		Advanced HIV disease stage	
		*N* = 40 (%)	OR (*P*)	*N* = 35 (%)	OR (*P*)	*N* = 64 (%)	OR (*P*)

AA	128 (81.0%)	38 (95.0%)	1 (reference)	33 (94.3%)	1 (reference)	60 (93.8%)	1 (reference)
AT	26 (16.5%)	2 (5.0%)	0.26, (0.09)	2 (5.7%)	0.30 (0.15)	2 (3.1%)	0.16 (0.01)
TT	4 (2.5%)	0 (0.0%)	NS	0 (0.0%)	NS	2 (3.1%)	1.07 (0.71)

*TLR7* rs179009 A/G genotypes	Healthy controls *N* = 158 (%)	Early HIV disease stage		Intermediate HIV disease stage		Advanced HIV disease stage	
		*N* = 40 (%)	OR (*P*)	*N* = 35 (%)	OR (*P*)	*N* = 64 (%)	OR (*P*)

AA	100 (63.3%)	38 (95.0%)	1 (reference)	33 (94.3%)	1 (reference)	60 (93.8%)	1 (reference)
AG	46 (29.1%)	2 (5.0%)	0.11 (0.001)	5 (5.7%)	0.03 (0.04)	3 (4.7%)	0.11 (≤0.001)
GG	12 (7.6%)	0 (0.0%)	NS	0 (0.0%)	NS	1 (1.6%)	0.14 (0.03)

*N* = umber of subjects; % = frequency of genotypes/alleles. The presence of AA for AT, TT for rs179008, AA for AG, GG for rs179009, and A allele for T and A allele for G was taken as a reference group for statistical analysis.

**Table 5:** Frequency distribution of *TLR7* polymorphisms (rs179008, *Gln11Leu* (*A/T*) and rs179009, IVS2-151 (A/G)) in tobacco and alcohol using HIV-infected individuals and nonusers.

*TLR7* rs179008 A/T genotypes	Tobacco users *N* = 23 (%)	Tobacco nonusers *N* = 122 (%)	*P* value	OR 95% CI

CC	19 (82.6%)	101 (82.8%)	1	Reference
CT	3 (13.0%)	17 (13.9%)	0.81	0.94 (0.20-3.89)
TT	1 (4.3%)	4 (3.3%)	0.70	1.33 (cornfield limits invalid)

*TLR7* rs179009 A/G genotypes	Tobacco users *N* = 23 (%)	Tobacco nonusers *N* = 86 (%)	*P* value	OR 95% CI

AA	15 (65.2%)	90 (73.8%)	1	Reference
AG	6 (26.1%)	21 (17.2%)	0.47	1.71 (0.52-5.49)
GG	2 (8.7%)	11 (9.0%)	0.75	1.09 (0.0-6.09)

*TLR7* rs179008 A/T	Alcohol users *N* = 23 (%)	Alcohol nonusers *N* = 127 (%)	*P* value	OR 95% CI

AA	20 (87.0%)	104 (81.9%)	1	Reference
AT	3 (13.0%)	18 (14.2%)	0.91	0.87 (0.18-3.55)
TT	0 (0.0%)	5 (3.9%)	—	NS

*TLR7* rs179009 A/G	Alcohol users *N* = 18 (%)	Alcohol nonusers *N* = 90 (%)	*P* value	OR (95% CI)

AA	18 (78.3%)	92 (72.4%)	1	Reference
AG	4 (17.4%)	23 (18.1%)	0.92	0.89 (0.23-3.17)
GG	1 (4.3%)	12 (9.4%)	0.68	0.43 (0.02-3.53)

*N* = number of subjects; % = frequency of genotypes/alleles. The presence of AA for AT, TT for rs179008, AA for AG, GG for rs179009, and A allele for T and A allele for G was taken as a reference group for statistical analysis.

## Data Availability

Data can be available through request by email.
